# Paclitaxel triggers molecular and cellular changes in the choroid plexus

**DOI:** 10.3389/fpain.2024.1488369

**Published:** 2024-11-25

**Authors:** Alemeh Zamani, Parisa EmamiAref, Lucie Kubíčková, Klaudia Hašanová, Ondřej Šandor, Petr Dubový, Marek Joukal

**Affiliations:** Department of Anatomy, Alemeh Zamani Research Group, Faculty of Medicine, Masaryk University, Brno, Czechia

**Keywords:** choroid plexus, paclitaxel, blood-CSF barrier, DAMPs, neuroinflammation, PINP

## Abstract

Paclitaxel is a widely used chemotherapeutic agent for treating various solid tumors. However, resulting neuropathic pain, often a lifelong side effect of paclitaxel, can limit dosing and compromise optimal treatment. The choroid plexus, located in the brain ventricles, spreads peripheral inflammatory reactions into the brain. Our study is the first to analyze the effects of paclitaxel on inflammatory alterations in the choroid plexus. We hypothesized that the choroid plexus could respond *directly* to paclitaxel and simultaneously be *indirectly* altered via circulating damage-associated molecular patterns (DAMPs) produced by paclitaxel application. Using immunohistochemical and Western blot analysis, we examined the levels of toll-like receptor 9 (TLR9) and formyl peptide receptor 2 (FPR2), along with the pro-inflammatory cytokines interleukin 6 (IL6) and tumor necrosis factor α (TNFα) in choroid plexus epithelial cells of male Wistar rats following paclitaxel treatment. Moreover, we utilized an *in vitro* model of choroid plexus epithelial cells, the Z310 cells, to investigate the changes in these cells in response to paclitaxel and DAMPs (CpG ODN). Our results demonstrate that paclitaxel increases TLR9 and FPR2 levels in the choroid plexus while inducing IL6 and TNFα upregulation in both acute and chronic manners. *In vitro* experiments further revealed that paclitaxel *directly* interacts with epithelial cells of the choroid plexus, leading to increased levels of TLR9, FPR2, IL6, and TNFα. Additionally, treatment of cells with CpG ODN, an agonist of TLR9, elicited upregulation of IL6 and TNFα. Our findings determined that paclitaxel influences the choroid plexus through both *direct* and *indirect* mechanisms, resulting in inflammatory profile alterations. Given the pivotal role of the choroid plexus in brain homeostasis, a compromised choroid plexus following chemotherapy may facilitate the spread of peripheral inflammation into the brain, consequently exacerbating the development of neuropathic pain.

## 1 Introduction

Paclitaxel, a commonly used and effective chemotherapeutic agent, can trigger toxic effects on peripheral nerves, often causing dose-dependent neuropathic pain (1). Paclitaxel-induced neuropathic pain (PINP), characterized by somatosensory dysfunction, negatively impacts patients’ quality of life, and currently, there are no preventative or therapeutic treatments available (2). Though we now recognize paclitaxel primary targets within the peripheral nervous system (2–4), underlying mechanisms of paclitaxel-induced neurotoxicity in the central nervous system (CNS) remain unclear.

The anterior cingulate cortex, involved in pain perception and modulation, displayed reactive astrocytes following paclitaxel application (5). These reactive astrocytes were associated with thermal hyperalgesia development, involving modulation of the glutamatergic and *γ*-aminobutyric acid systems (5, 6). A growing body of evidence also suggests that paclitaxel upregulates pro-inflammatory mediators in dorsal root ganglia and the spinal cord dorsal horn, often associated with activated macrophages (7, 8). The activation of peripheral and central glial cells, along with the upregulation of pro-inflammatory cytokines, seems to be implicated in the development of PINP (9–11).

Furthermore, research demonstrates distal axonal degeneration subsequent to the release of pro-inflammatory cytokines and chemokines following paclitaxel treatment (12, 13). Axonal damage leads to increased circulating damage-associated molecular patterns (DAMPs), which are sensed by the pattern recognition receptors (PRRs), key mediators of the innate immune system (14).

The choroid plexus, located in the brain's ventricles, comprises fenestrated capillaries and a monolayer of epithelial cells by which the blood-cerebrospinal fluid (CSF) barrier is formed. While the tightly connected epithelial cells selectively limit and regulate the passage of substances into and out of the brain, fenestrated capillaries permit easy passage of blood components, including circulating immune cells and DAMPs. Additionally, the choroid plexus is presumably the only region of the CNS in which systemically administrated paclitaxel has been detected (15).

The presence of toll-like receptor 4 (TLR4), the specific receptor for paclitaxel (16), as well as other TLRs (TLR1–7 and TLR9–10), has been reported in the epithelial cells of the choroid plexus across different species. This suggests that the choroid plexus has the capacity to detect various DAMPs and produce pro-inflammatory cytokines such as interleukin (IL)-6 and tumor necrosis factor (TNFα) (17–19). Research has evidenced that TLRs ligands present in the bloodstream (DAMPs) stimulate TLRs located in the choroid plexus, thereby transferring immune signals to the brain (20–23). Nonetheless, the regulatory mechanisms of TLRs in the choroid plexus during systemic inflammation are yet to be defined. Since the formyl-peptide receptor 2 (FPR2), a G protein-coupled receptor activated by a wide range of ligands is expressed in monocytes, macrophages, astrocytes (24), and choroid plexus epithelial cells (25), we speculated that the choroid plexus epithelial cells expressing a range of immunoreceptors might be involved in spreading peripheral paclitaxel-induced inflammation into the CNS.

Understanding the potential role of the choroid plexus in the spread of inflammatory responses to the CNS during paclitaxel treatment involves considering two possible scenarios. First, as referenced above, paclitaxel could access the choroid plexus and *directly* alter its function, activating the PRRs of epithelial cells and inducing upregulation of cytokines. In a second scenario, circulating DAMPs produced by paclitaxel treatment may access the choroid plexus via its fenestrated capillaries, thereby causing an *indirect* inflammatory profile alteration.

Ascertaining the validity of aforementioned scenarios, we used an established animal model of PINP (26) and an *in vitro* model of the choroid plexus epithelial cells (27). We assessed TLR9 and FPR2 levels in the choroid plexus of the *in vivo* model, exploring alterations that may result from *direct*, *indirect*, or combined paclitaxel effects. Accordingly, immunohistochemical and Western blot analyses were used to investigate the changes in these PRRs and pro-inflammatory cytokines IL6 and TNFα.

To better understand the mechanisms of the *direct* and *indirect* effects of paclitaxel on the choroid plexus, we used the *in vitro* model, Z310 cells, to evaluate cellular immune responses to both paclitaxel and DAMPs. In these experiments, we investigated whether TLR9 and FPR2 activation is induced *directly* by paclitaxel or *indirectly* by their respective agonists.

## 2 Materials and methods

### 2.1 *In vivo* investigation of paclitaxel effects on the choroid plexus

#### 2.1.1 Animals and surgical procedure

All experiments with animals respected the European Convention for the Protection of Vertebrate Animals Used for Experimental and Other Scientific Purposes. The Animal Investigation Committee of the Faculty of Medicine Masaryk University, Brno, Czech Republic, approved the protocol.

Experiments involved 74 adult male rats (Wistar, 200–250 g; Masaryk University Animal Breeding Facility). The animals were kept on a 12 h light/dark cycle (lights on at 06:00) with food and water available *ad libitum*.

We employed a previously established animal model of paclitaxel-induced neuropathy (26). Experimental rats were randomly assigned to 2 experimental groups: Paclitaxel-treated animals (*n* = 28 in total) and vehicle-administrated animals (*n* = 28 in total). Animals without treatment were used as naïve control (*n* = 18). Paclitaxel (Sigma–Aldrich; 2 mg/kg) or its vehicle (Cremophor EL and 95% EtOH in 1:1 ratio, Sigma) were injected intraperitoneally (IP) in rats on alternate days (days 1, 3, 5, and 7; cumulative dose of 8 mg/kg).

Rats of the paclitaxel and the vehicle group were sacrificed after 1, 7, 14, and 21 days. Three animals per experimental group underwent immunohistochemical (IHC) analysis, and four underwent Western blot analysis.

#### 2.1.2 Tissue processing and immunohistochemical staining

Animals used for IHC were eliminated via CO_2_ inhalation and perfused transcardially with phosphate-buffered saline (PBS; pH 7.4), followed by 500 ml of Zamboni's fixative (28). Brains were removed and immersed in Zamboni's fixative for 3 days at 4°C. Brains were then washed in 10% sucrose and embedded in the Tissue-Tek OCT compound (Miles; Elkhart, IN). Serial coronal cryostat sections (20 µm) were cut using a Leica 1800 cryostat (Leica Microsystems, Wetzlar, Germany) and mounted onto chrome-alum-covered microscopic slides for staining.

Brain sections were washed with PBS containing 0.3% bovine serum albumin and 0.1% Tween-20, treated with 3% normal goat serum for 30 min, and incubated with primary antibodies (Table 1) in a humid chamber at room temperature (21–23°C). Next, sections were rinsed with PBS for 10 min and incubated with Affinity purified CY5-conjugated goat anti-mouse or goat anti-rabbit secondary antibodies (1:100) at room temperature for 90 min. Sections were further rinsed in PBS, stained with Hoechst 33342 (Sigma; St. Louis, MO) to detect cell nuclei, and mounted in a Vectashield aqueous mounting medium (Vector Laboratories; Burlingame, CA). Control sections were incubated in parallel without the primary antibody.

**Table 1:** List of antibodies used for immunostaining.

Name	Clonality	Dilution	Incubation	Catalog number
TLR9	Rabbit polyclonal	1:100	Overnight, RT	(Santa Cruz Biotechnology Cat# sc-52966, RRID: AB_793207)
FPR2	Rabbit polyclonal	1:100	Overnight, RT	(Novus Cat# NLS1878, RRID:AB_2294156)
IL6	Rabbit polyclonal	1:100	Overnight, RT	(Novus Cat# NB600-1131, RRID:AB_10001997)
TNF*α*	Rabbit polyclonal	1:1,000	Overnight, RT	(Abcam Cat# ab66579, RRID:AB_1310759),

#### 2.1.3 Western blot analysis

For Western blot analysis, choroid plexus from both lateral and fourth ventricles were removed and flash-frozen in liquid nitrogen. Samples were kept at −80°C until further processing. Choroid plexus tissue was homogenized in RIPA buffer containing protease inhibitor cocktail and phosphatase inhibitor (Complete Mini; Roche Diagnostics, Basel, Switzerland) and centrifuged at 10,000 g for 15 min at 4°C. Protein concentrations of the supernatants were assessed using the BioRad protein assay kit. Next, proteins were separated by SDS-polyacrylamide gel electrophoresis and transferred to polyvinylidene difluoride membranes by electroblotting (BioRad). Blots were blocked using 5% nonfat dried milk in Tris-buffered saline (TBS) with Tween 20 (3.2 mM Na2HPO4, 0.5 mM KH2PO4, 1.3 mM KCl, 135 mM NaCl; pH 7.4) for at least 1 h and incubated with primary antibodies overnight (Table 2). Vinculin (1:10,000) was used as an internal control. Blots were washed in TBST and incubated with peroxidase-conjugated anti-mouse or anti-rabbit IgG (1:5,000; Cell Signaling) at room temperature for 2 h. Protein bands were visualized by the enhanced chemiluminescence kit (SuperSignal™ West Pico PLUS; ThermoFisher) and the BioRad ChemiDocTM XRS + System and analyzed using densitometry image software (Image Lab; BioRad). Experiments were repeated three times.

**Table 2:** List of antibodies used for western blot.

Name	Clonality	Dilution	Incubation	Catalog number
Vinculin	Rabbit monoclonal	1:10,000	Overnight, 4°C	(Abcam Cat# ab129002, RRID:AB_11144129),
TLR9	Mouse monoclonal	1:250	Overnight, 4°C	(Santa Cruz Biotechnology Cat# sc-52966, RRID:AB_793207)
FPR2	Rabbit polyclonal	1:1000	Overnight, 4°C	(Novus Cat# NLS1878, RRID:AB_2294156)
IL6	Rabbit polyclonal	1:500	Overnight, 4°C	(Novus Cat# NB 600-1131, RRID:AB_577984)
TNFα	Rabbit polyclonal	1:500	Overnight, 4°C	(Abcam Cat# ab66579, RRID:AB_1310759)
HRP-linked	Rabbit	1:5,000	1–2 h, RT	Cell signalling #7074
HRP-linked	Mouse	1:5,000	1–2 h, RT	Cell signalling #7076

### 2.2 *In vitro* experiments to explore the *direct* and/or *indirect* effects of paclitaxel

#### 2.2.1 Z310 cell culture

Immortalized rat choroidal epithelial Z310 cells and the cell culture protocol were kindly provided by Prof. Wei Zheng [Purdue University, USA; (27)]. Concisely, Z310 cells were grown to 80%–90% confluence in the culture flask in Dulbecco's Modified Eagle's Medium (DMEM-F12) under a 5% CO_2_ atmosphere at 37°C. The medium was supplemented with 10% fetal bovine serum, epidermal growth factor (EGF), and antibiotics (penicillin, streptomycin, and gentamicin). Accutase was used to detach Z310 cells for cell passage.

#### 2.2.2 Cell treatment

Aliquots (0.1 ml; 4.0 × 10^4^ cells) of cell suspensions were added to 13 mm glass coverslips (Deckgläser, Germany) in 12- or 24-well plates. After cell attachment, 1 ml of medium was added to each well. Prior to cell seeding, the coverslips were washed with 97% ethanol and pre-coated with 0.05% collagen for 3 h.

When the formation of the barrier-like structure in culture was confirmed using a light microscope, Z310 cells were incubated with 20nM paclitaxel/DMSO for 24 h. Paclitaxel concentration was first determined by MTT test. Control cells were incubated with only DMSO (<0.01%) for 24 h.

Further, by activating the PRRs in cultured cells using their respective agonists, we investigated the possible *indirect* effects of paclitaxel on the choroid plexus. Administrated agonists included CpG ODN and N-formyl-methionyl-leucyl-phenylalanine (fMLP) for TLR9 and FPR2, respectively.

Prior to the agonist administration, 4.0 × 10^4^ cells were seeded in 12-well plates, as noted above. Next, fMLP/DMSO (20 μM) and CpG ODN/H_2_O (2.5 μM) were added to the culture for 24 h. Equivalent volumes of DMSO (<0.01%) or H_2_O were used in the controls.

We first performed the Live/Dead assay with different concentrations of the aforementioned agonists ranging from 1 µM–100 µM to select the lowest effective concentration with cell viability of 75%.

#### 2.2.3 Immunocytochemical staining (ICC)

For cultured cell staining, at the end of the described treatments, cells were fixed with 4% paraformaldehyde in PBS for 20 min, washed three times with PBS, and permeabilized with cold methanol: acetone (1:1). Cells were immunostained overnight with appropriate primary antibodies (Table 1). The immunocytochemical reactions were visualized using affinity-purified CY5-conjugated goat anti-rabbit antibody (1:100; Millipore) for 90 min at room temperature. Control cells were incubated without the primary antibody. Cell nuclei were stained with DAPI, and slides were mounted in an aqueous mounting medium (Vectashield; Vector Laboratories, Burlingame, CA, USA).

#### 2.2.4 Western blot analysis

Z310 cells were harvested and dissolved in RIPA buffer with protease inhibitor cocktail and phosphatase inhibitor (Complete Mini; Roche Diagnostics, Basel, Switzerland) and centrifuged at 10,000 g for 15 min at 4°C. The protein concentration was determined using the BioRad protein assay kit. Proteins were resolved by SDS-PAGE and transferred onto a polyvinylidene difluoride membrane. After blocking with 5% nonfat dry skim milk for 1 h at room temperature, membranes were incubated overnight at 4°C with primary antibodies (Table 2). Following rinses using TBST, the membranes were incubated for 1 h with HRP-conjugated secondary antibodies (Table 2) at room temperature. Protein bands were visualized using enhanced chemiluminescence (SuperSignal™ West Pico PLUS; ThermoFisher) and the BioRad ChemiDocTM XRS + System. Bands were normalized to vinculin and analyzed using densitometry image software (Image Lab; BioRad). Experiments were repeated three times.

### 2.3 Image analysis of immunofluorescence

An epifluorescence microscope (Nikon Eclipse NI-E Motorized Microscope System) equipped with a stabilized power supply for the lamp housing and a Nikon DS-Ri1 camera (Nikon, Prague, Czech Republic) was used for image acquisitions. Images of brain sections containing the choroid plexus of lateral ventricles or Z310 cells were acquired using identical parameters of the camera, optics, and lamp, as well as exposure conditions. Fluorescence intensity was quantified using ImageJ image analysis software (Rasband, W.S., ImageJ, U. S. National Institutes of Health, USA). Intensity threshold was objectively set based on the mean intensity of control samples, ensuring that only areas with significant staining were highlighted. Integrated density was measured for each sample and expressed as means ± SD.

### 2.4 Statistical analyses

Statistical differences between the two groups were determined by Student's *t*-test, and multiple groups were analyzed by one-way analysis of variance (*p* < 0.05). All statistical analyses were performed using STATISTICA 9.0 software (StatSoft, Inc., Tulsa, OK, USA).

## 3 Results

### 3.1 TLR9 and FPR2 levels in choroid plexus increased following paclitaxel treatment

**Figure 1:**
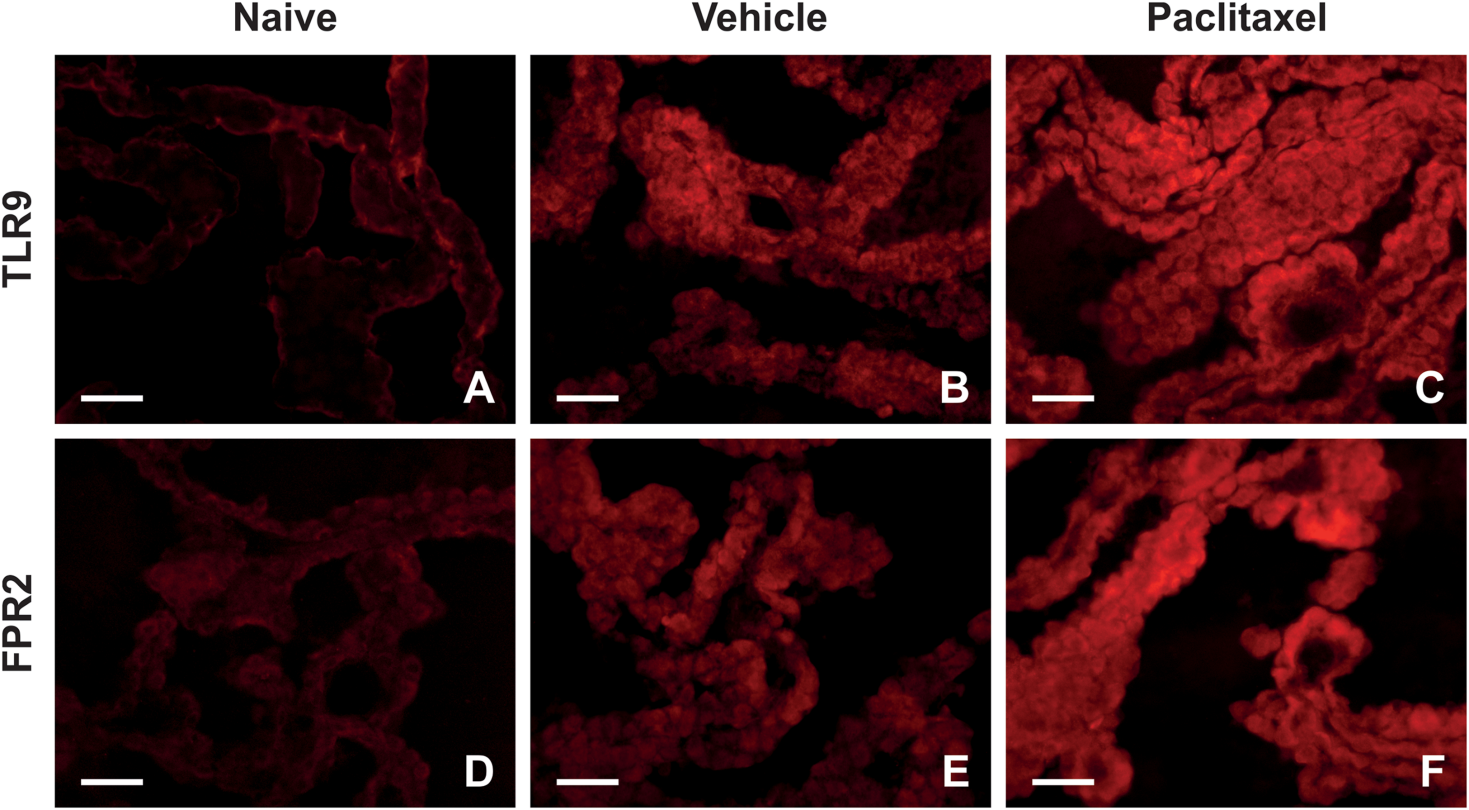
Representative pictures indicating localization and levels of TLR9 **(A–C)** and FPR2 **(D–F)** immunofluorescence in the choroid plexus of naïve, vehicle, and paclitaxel rats on day 1 after the last injection. Scale bars = 50 μm.

Before addressing PRRs level alteration in the choroid plexus, we examined the localization of these proteins in the choroid plexus of rats using immunofluorescence (IF) staining. Immunostaining of TLR9 and FPR2 proteins in the choroid plexus of naïve, vehicle, and paclitaxel-treated rats is illustrated in Figure 1. Distinct TLR9 immunopositivity was observed in the plasma membrane, with diffuse immunofluorescence in the cytoplasm (Figures 1A–C). In contrast, FPR2-IF was predominantly diffused in the cytoplasm of choroid plexus epithelial cells (Figures 1D–F).

**Figure 2:**
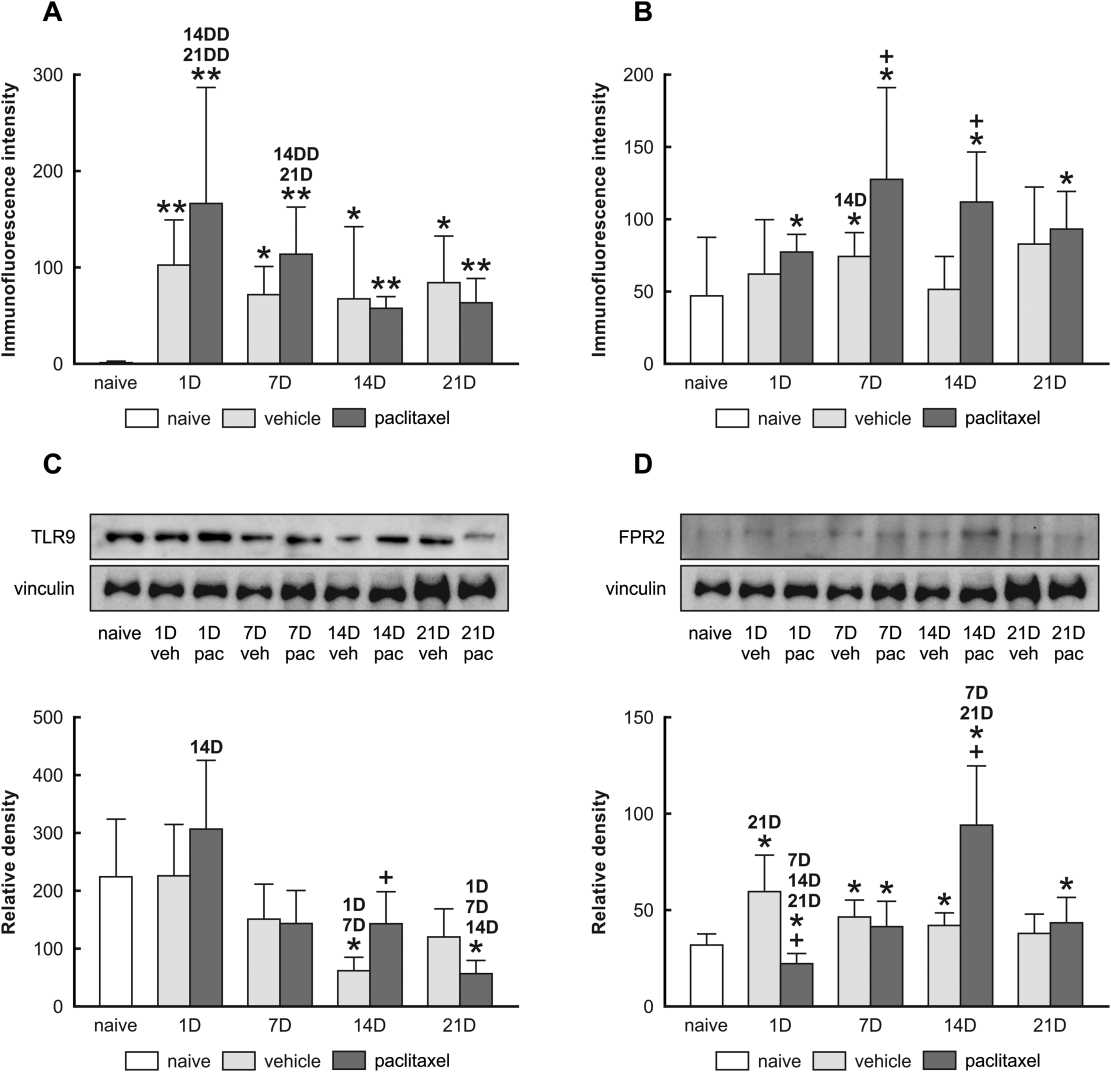
Semi-quantitative analysis of TLR9 and FPR2 immunofluorescence intensities in choroid plexus epithelial cells of naïve, vehicle, and paclitaxel rats 1, 7, 14, and 21 days after the last IP injection. The integrated density (±SD) of TLR9 and FPR2 immunofluorescence in choroid plexus epithelial cells is shown in panels **(A,B)** respectively. Representative blots (upper panel) and Western blot analysis of TLR9 **(C)** and FPR2 **(D)** protein bands after normalization to vinculin are displayed in the lower panel. *Indicates significant difference (*p* < 0.05) when compared with choroid plexus from naïve rats. **Indicates significant difference (*p* < 0.01) when compared with choroid plexus from naïve rats. +Indicates significant difference (*p* < 0.05) when compared with choroid plexus from the vehicle group. 1D,7D,14D, and 21D indicate significant differences (*p* < 0.05) with their corresponding days in the same group. 14DD and 21DD indicate significant differences (*p* < 0.01) with their corresponding days in the same group.

Semiquantitative analysis determined that levels of TLR9-IF intensities were significantly increased when examined one day after the last IP injection of both paclitaxel and vehicle, compared with naïve (Figure 2A). As survival time increased, the intensities of TLR9-IF decreased with the time of survival yet remained higher than that of the naïve The FPR2-IF intensities were significantly higher than naïve at all survival times after paclitaxel application, with a peak at 7D, when the intensity was also significantly higher than naïve control after vehicle application. In addition, the FPR2-IF intensities were significantly higher after paclitaxel than after vehicle application at 7D and 14D (Figure 2B). Although both immunohistochemical and Western blot analyses showed changes in TLR9 after treatment with both vehicle and paclitaxel, the results of these methods did not correlate with each other exactly (Figure 2C). Differences were also found between the results of semiquantitative analysis of FPR2-IF and Western blot analysis, where the highest FPR2 level was detected in the paclitaxel group on day 14, with significant increases also on days 7 and 21 compared to the naïve group (Figure 2D).

### 3.2 Pro-inflammatory cytokine levels increased following paclitaxel application

**Figure 3:**
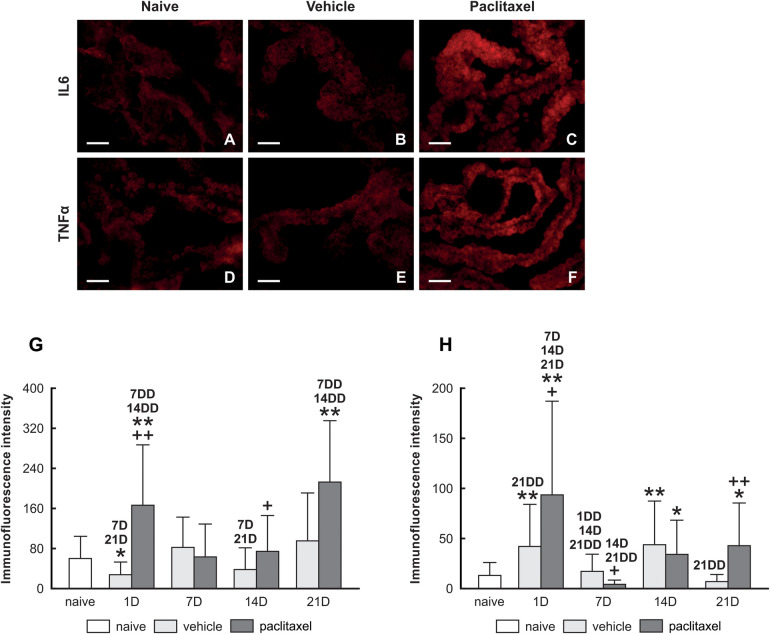
Upper panel: representative pictures illustrating IL6 and TNF*α* immunofluorescence intensities of IL6 **(A–C)** and TNFα **(D–F)** in choroid plexus epithelial cells of naïve, vehicle, and paclitaxel groups. Scale bars = 50 μm. Lower panel: Semiquantitative analysis Immunoquantification of IL6 and TNF*α* in choroid plexus epithelial cells of naïve, vehicle, and paclitaxel rats 1, 7, 14, and 21 days after the last IP injection. The integrated density (±SD) of IL6 and TNFα immunofluorescence in choroid plexus epithelial cells is illustrated in panels **(G,H)**, respectively. *Indicates significant difference (*p* < 0.05) when compared with choroid plexus from naïve rats. **Indicates significant difference (*p* < 0.01) when compared with choroid plexus from naïve rats. +Indicates significant difference (*p* < 0.05) when compared with choroid plexus from the vehicle group. ++Indicates significant difference (*p* < 0.01) when compared with choroid plexus from the vehicle group. 7D,14D, and 21D indicate significant differences (*p* < 0.05) with their corresponding days in the same group. 1DD, 7DD, 14DD, and 21DD indicate significant differences (*p* < 0.01) with their corresponding days in the same group.

PRR increased levels can be associated with immune responses and induce pro-inflammatory cytokines. We examined the levels of IL6 (Figures 3A–C) and TNFα (Figures 3D–F) proteins in choroid plexus epithelial cells of naïve, vehicle, and paclitaxel groups. Our immunoquantification results illustrate the upregulation of IL6 and TNFα cytokines in the paclitaxel group compared with vehicle and naïve animals examined 1 day after the last dose (Figures 3G,H). We did not record any increase in the level of cytokines when examining the choroid plexus at 7D and 14D. However, an increase in the level of IL6 and TNFα cytokines was observed at 21D.

### 3.3 Unraveling the *direct* and *indirect* components of paclitaxel effects on choroid plexus

Our findings from the *in vivo* model recorded acute and chronic inflammatory responses in the choroid plexus after paclitaxel treatment. Therefore, we employed an *in vitro* model of the choroid plexus, the Z310 cells, aiming to understand whether the acute response could arise from the *direct* interaction of paclitaxel with the choroid plexus epithelial cells. We therefore exposed Z310 cells to paclitaxel and examined the changes in TLR9 and FPR2, as well as subsequent cytokines levels.

**Figure 4:**
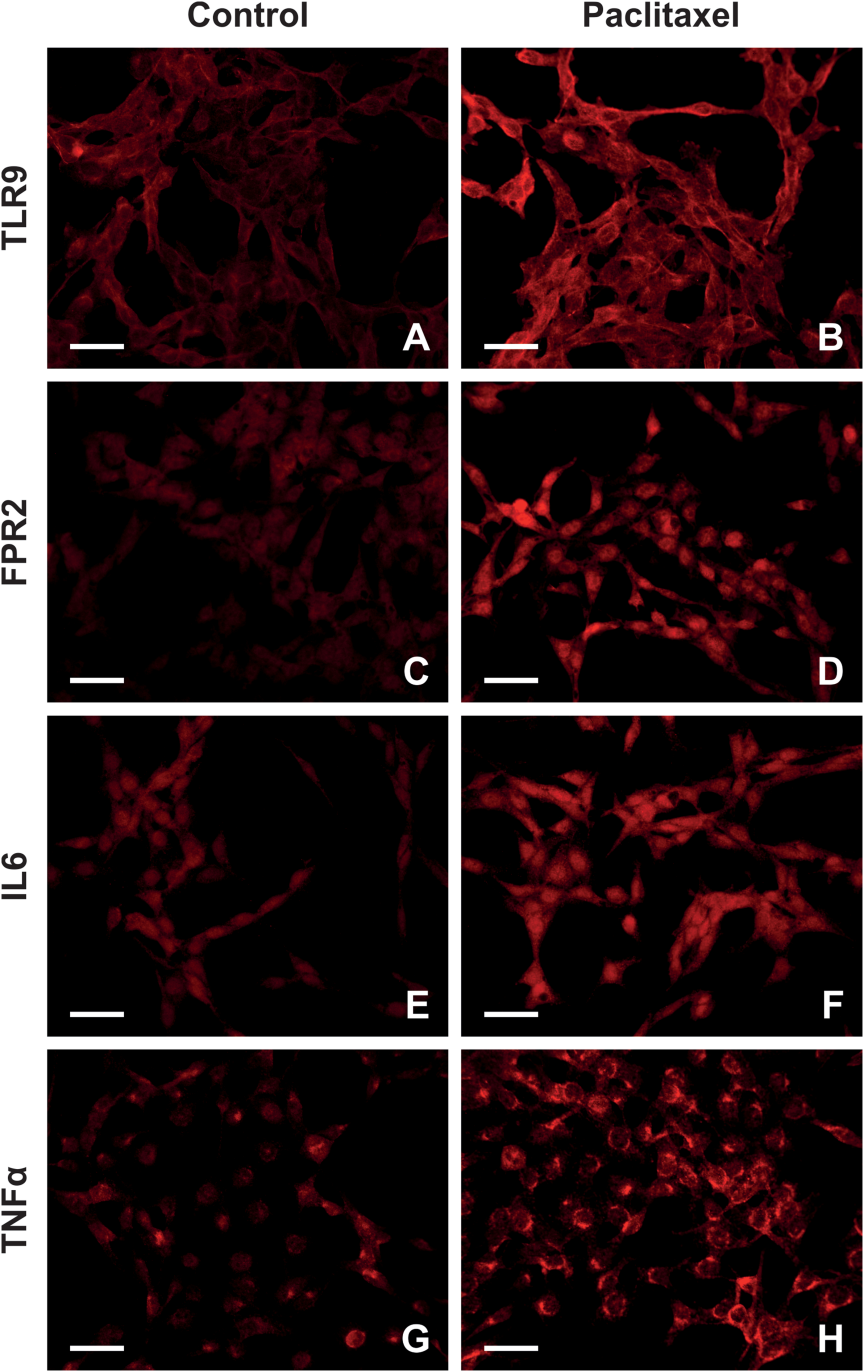
Representative images highlighting TLR9, FPR2, IL6, and TNFα expression in Z310 cells after paclitaxel administration. Panel **(A–H)** illustrates TLR9, FPR2, IL6, and TNFα expression patterns in control and paclitaxel-treated cells.

**Figure 5:**
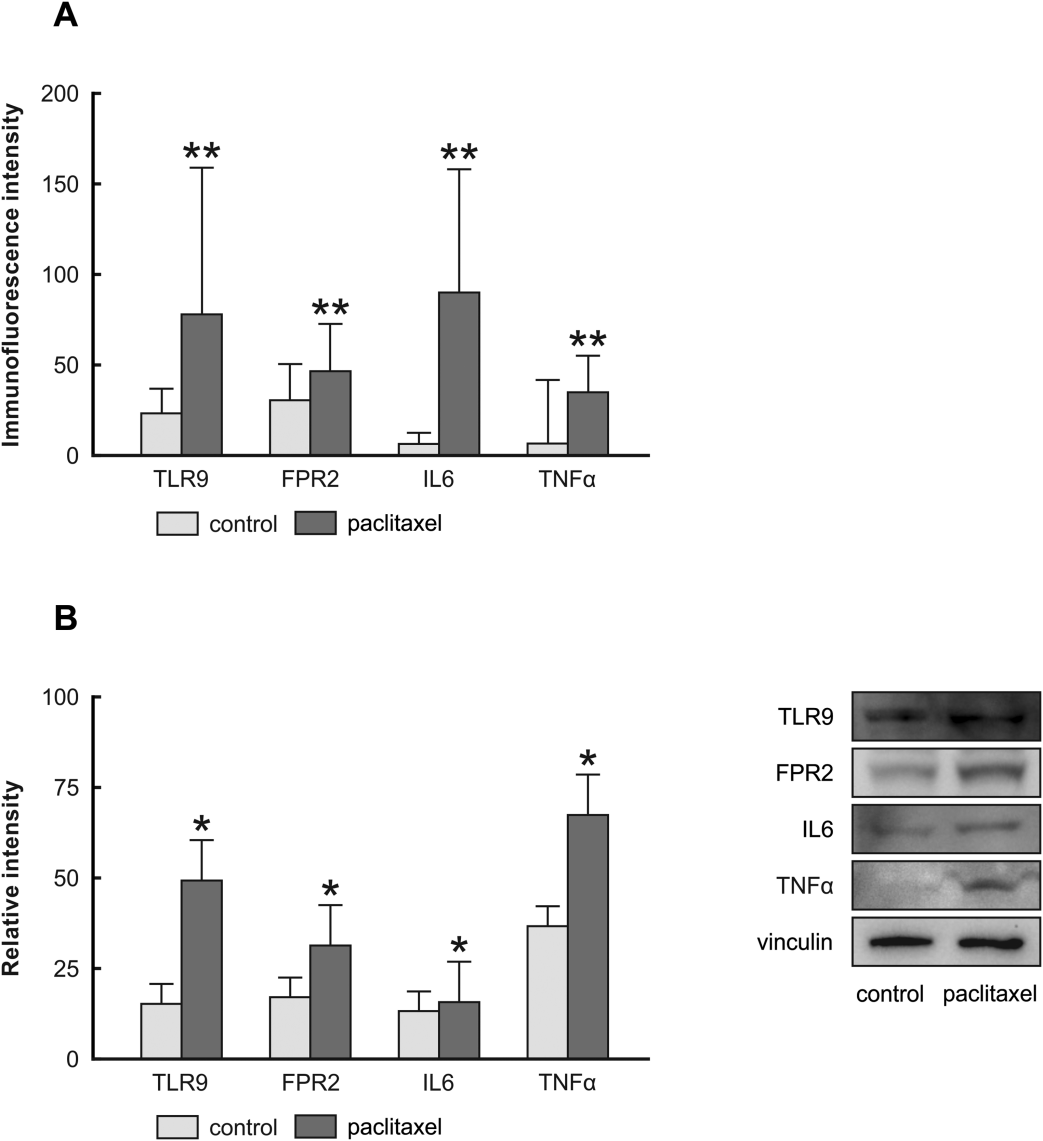
Immunoquantification of TLR9, FPR2, IL6, and TNFα in Z310 cells 24 h after paclitaxel treatment. The integrated density (±SD) of TLR9, FPR2, IL6, and TNFα immunofluorescence in Z310 cells is evidenced in panel **(A)** Representative blots (right panel) and densitometry of TLR9, FPR2, IL6, and TNFα protein bands after normalization to vinculin are in the left panel **(B)**.

Figure 4 depicts the presence of TLR9, FPR2, IL6, and TNF*α* in the cytoplasm and plasma membrane of Z310 cells. Similar to results of *in vivo* experiments, paclitaxel administration for 24 h caused upregulation of TLR9 and FPR2 in Z310 cells, as well as the pro-inflammatory cytokines IL6 and TNFα (Figure 5A). The upregulation of PRRs and pro-inflammatory cytokines was confirmed by Western blot (Figure 5B).

**Figure 6:**
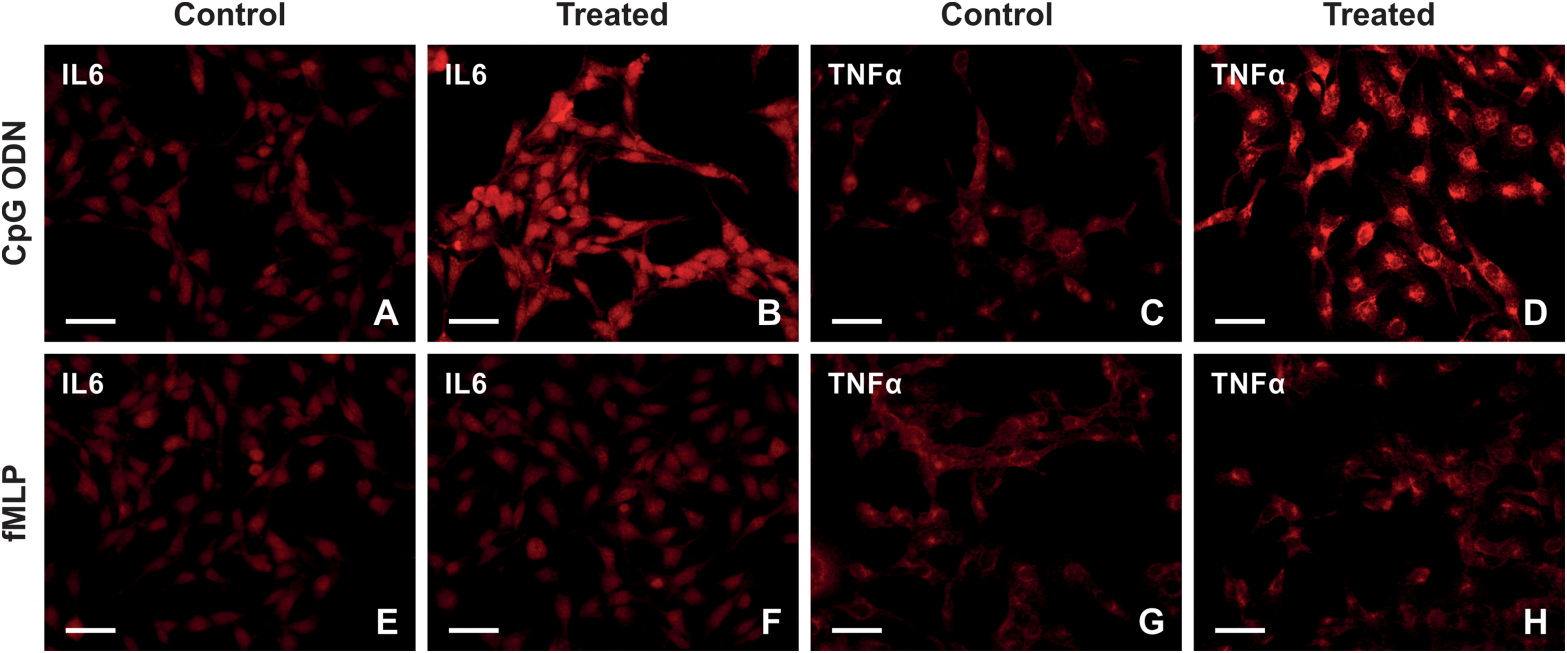
Representative images illustrating immunostaining of IL6 and TNFα in Z310 cells treated with CpG ODN, fMLP, and their controls. **(A–D)** The upper row shows the expression of IL6 and TNFα in Z310 cells treated with CpG ODN or control **(E–H)**. The lower row illustrates the expression of IL6 and TNFα in treated cells with fMLP, compared with its control. scale bars = 50 μm.

**Figure 7:**
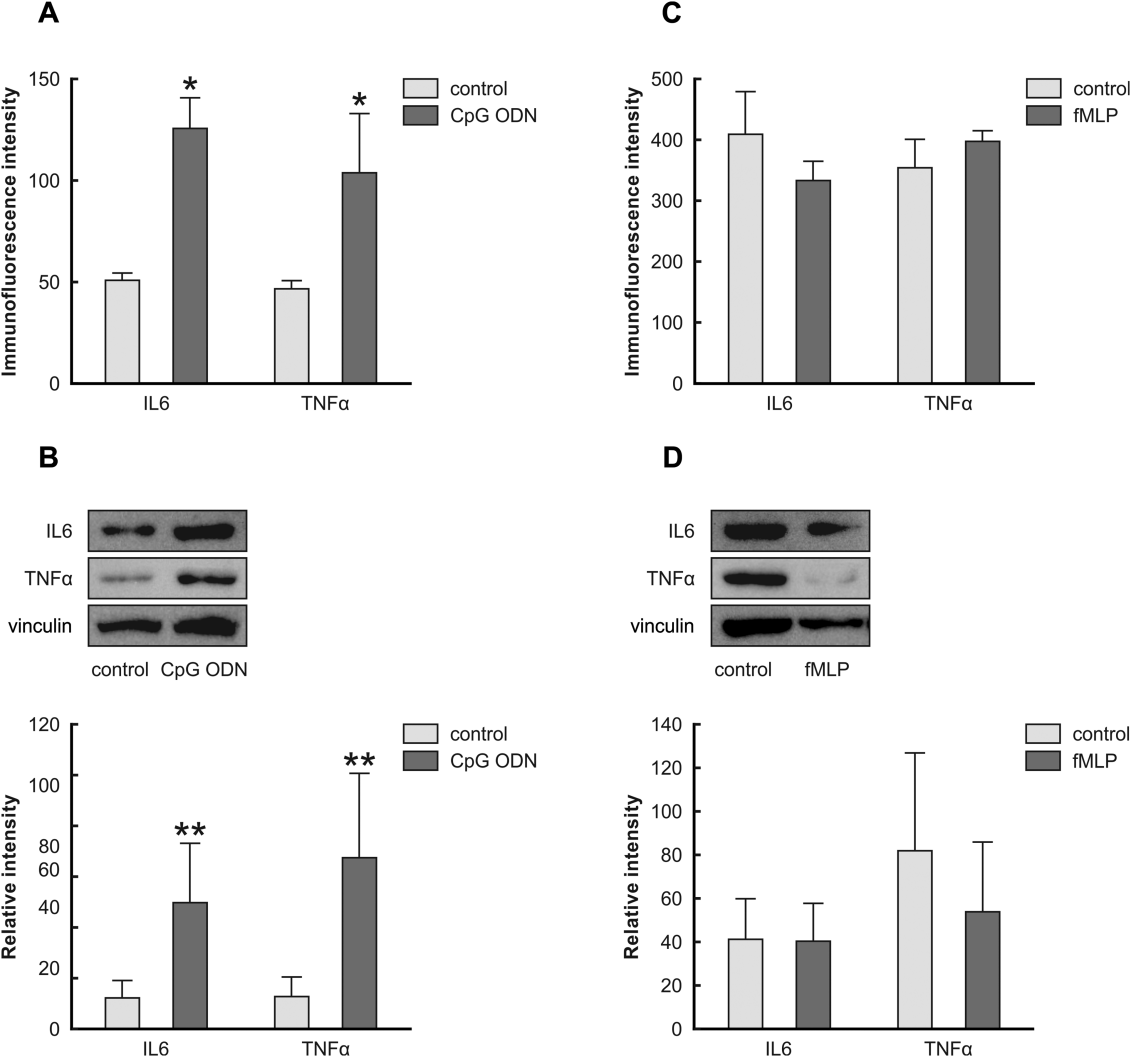
Effects of CpG ODN and fMLP stimulation on the levels of IL6 and TNFα in Z310 cells. Protein levels of IL6 and TNFα in Z310 cells treated with 2.5 µM of CpG ODN (TLR9 agonist) and 20 µM fMLP (FPR2 agonist) were analyzed using ICC and Western blots. Panel **(A)** shows the immunoquantification of IL6 and TNFα in cells treated with CpG ODN for 24 h. Results of densitometric measurements of bands from triplicate Western blot analysis normalized to the housekeeping protein vinculin are depicted in the graphs in panel **(B)** Graph **(C)** denotes the immunoquantification of IL6 and TNFα in cells treated with fMLP for 24 h. Densitometric measurements of bands from triplicate Western blot analysis normalized to the vinculin are shown in panel **(D)** graphs. Results are characterized as means ± SEM. *Indicates *p* < 0.05, and **indicates *p* < 0.01 compared to the control.

Our *in vivo* results indicated the choroid plexus epithelial cells reacted to the paclitaxel treatment 21 days after the last injection, and we speculated that the chronic reaction is mediated secondarily by DAMPs present in choroid plexus stroma; hence, paclitaxel's *indirect* effect. To test this, we exposed Z310 cells to CpG ODN and fMLP, agonists of TLR9 and FPR2. Figures 6A–H illustrates the expression pattern of IL6 and TNFα in Z310 cells when treated with CpG ODN or fMLP and their controls. Our ICC and Western blot data indicate that CpG ODN caused the upregulation of the pro-inflammatory cytokines IL6 and TNF*α* (Figures 7A,B). However, we did not detect any significant changes in IL6 and TNFα levels when cells were treated with fMLP (Figures 7C,D).

## 4 Discussion

**Figure 8:**
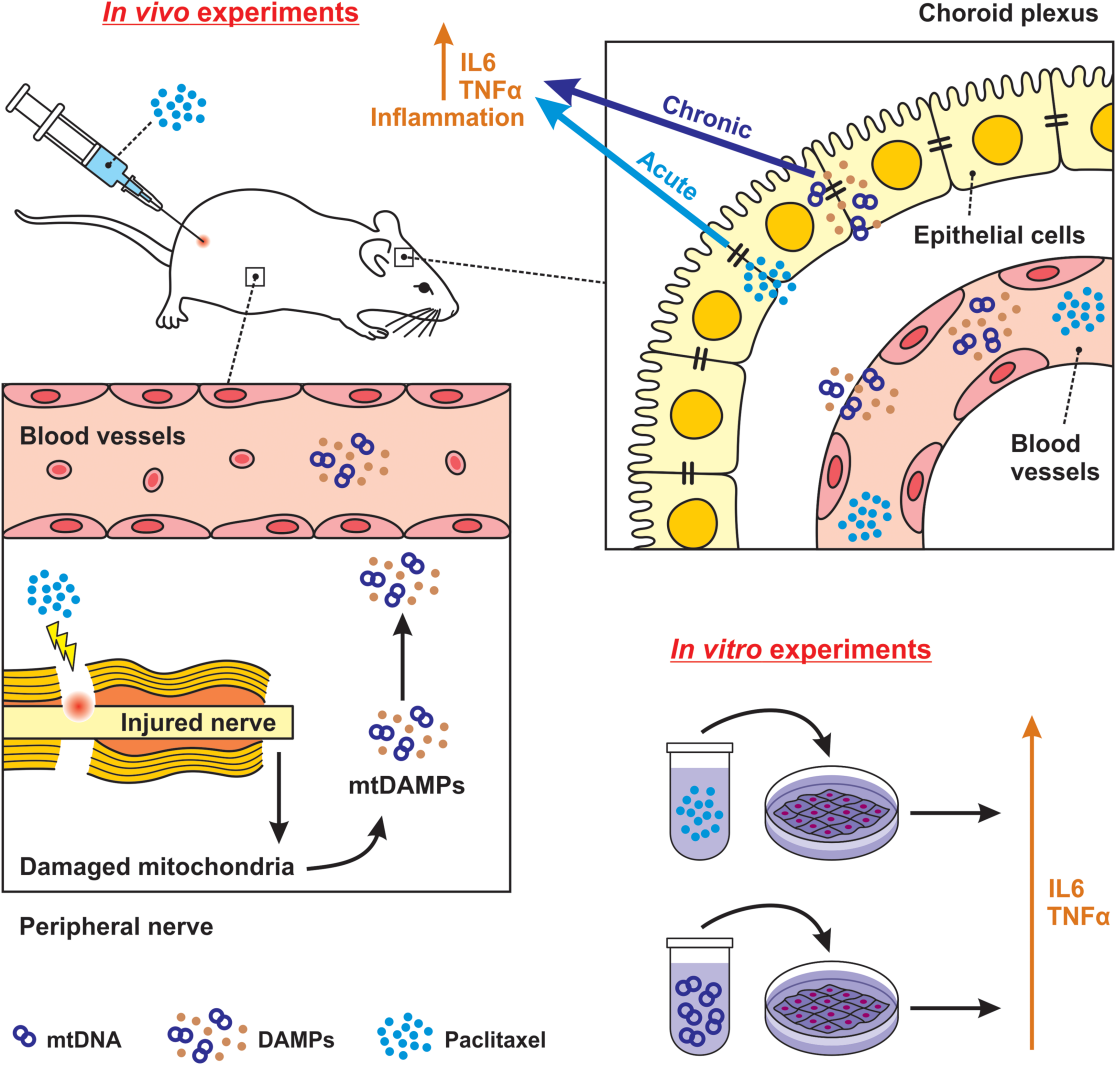
Schematic illustration of possible *direct* and *indirect* effects of paclitaxel on choroid plexus. Using an *in vivo* model, we determined that paclitaxel could promote an immediate inflammatory response in choroid plexus epithelial cells. The effect is defined as acute and *direct*, which was also confirmed via paclitaxel-elevation of TLR9 and FPR2 levels and subsequent upregulation of IL6 and TNFα cytokine in the *in vitro* model. We further clarified that the secondary inflammatory response in the choroid plexus, observed 21 days after the last paclitaxel injection, may be attributed to circulating DAMPs induced by paclitaxel treatment. Our *in vitro* finding confirmed the elevation of TLR9 by its agonist CpG ODN in choroidal epithelial cells. We characterize this as an *indirect* and chronic effect.

Our study is the first to provide evidence that paclitaxel treatment increases the levels of TLR9 and FPR2 proteins associated with the induction of both acute and chronic inflammatory responses in the choroid plexus (Figure 8). We used an established model of paclitaxel-induced neuropathic pain (27), in which the application of a cumulative dose of 8 mg/kg of paclitaxel induced thermal-hyperalgesia, mechano-allodynia, mechano-hyperalgesia, and cold-allodynia, reflecting the neuropathic pain behaviors relevant to our investigation.

This information is crucial to consider, as it indicates that paclitaxel treatment may pose risks to the brain despite its inability to cross the blood-brain barrier (29). A plurality of multiple evidence determines that the choroid plexus, because of its unique structure and significant effect on the CSF composition, is capable of linking the peripheral to CNS inflammation (18, 30, 31).

However, to our knowledge, no study has implicated the choroid plexus in paclitaxel-induced neurotoxicity. Based on our results, in addition to linking the peripheral inflammation to the CNS (responding to DAMPs produced by the action of paclitaxel on peripheral nerves), the choroid plexus importance is manifested in paclitaxel *directly* causing changes in the choroid plexus inflammatory profile.

Gaining insight into the precise interaction mechanism between paclitaxel and the choroid plexus could enhance chemotherapeutic strategies, preventing alterations to the choroid plexus that might otherwise compromise the integrity of the blood-CSF barrier (31). In addition, such changes in the choroid plexus could influence the CSF composition, potentially affecting inflammatory responses in other brain regions.

In the peripheral nervous system, altered ion channels and axonal transport, mitochondrial dysfunction, and immune responses subsequent to damaged nerve fibers caused by paclitaxel have been observed (2, 3, 11). The dorsal root ganglia are identified as a primary target for paclitaxel-induced neurotoxicity, attributed to their vulnerability owing to incomplete protection by the blood-nerve barrier. Therefore, paclitaxel accumulation in this structure has been linked with the infiltration of immune cells and activation of maladaptive inflammation (13, 32). Research indicates that the paclitaxeĺs mitotoxic effect specifically targets sensory neurons in the dorsal root ganglia, leading to the onset of painful peripheral neuropathy (33). An “on-wire” pathway could be involved in transmitting pain information via the sensory pathway to the center of the CNS.

In the central nervous system, paclitaxel activates the astrocytes of the anterior cingulate cortex, which involves pain perception and modulation (5). However, Lesser and colleagues note that following a systemic paclitaxel administration (0.3 mg/kg), no paclitaxel was distributed in the brain parenchyma, spinal cord, dorsal root ganglion, and peripheral nerve, whereas paclitaxel was detected in the choroid plexus and CSF (15). Considering the evidence, does limited distribution of paclitaxel in the nervous system with its long-lasting and irreversible peripheral neuropathy (4, 34) involve a “wire-less” pathway for the spread of pro-inflammatory mediators?

Building upon this speculation, we investigated the effects of paclitaxel on the singular structure of the brain, where paclitaxel had been detected and, similar to the dorsal root ganglia, fenestrated capillaries are present. We hypothesized that paclitaxel might not only *directly* induce cellular and molecular alterations in the choroid plexus but also trigger an immune response in the choroid plexus through the subsequent release of DAMPs into the bloodstream (*indirect* effect). Either scenario could potentially initiate and propagate an inflammatory cascade into the brain, thereby contributing to PINP development (22).

In this study, using an *in vivo* model, we observed that IP injection of paclitaxel induced the upregulation of TLR9 and FPR2 in the epithelial cells of rat choroid plexus (Figure 2). This was correlated with an acute inflammatory response, as we recorded IL6 and TNFα levels increase as early as the first day following the last paclitaxel injection (Figures 3G,H).

Research has evidenced TLRs involvement and its subsequent activation of different inflammatory pathways in the development of chemotherapy-induced neuropathic pain. Significantly, upregulation of TLR2, 3, 4, and 9 in dorsal root ganglia of paclitaxel-treated animals has been reported (35–37). On the other hand, a recent study showed that paclitaxel treatment did not alter FPR2 mRNA expression in the dorsal root ganglia, which contrasts our findings in the choroid plexus (38). While further research is required, this implies a more intricate sequence of events within the choroid plexus that regulates the inflammatory response to paclitaxel.

The immediate inflammatory response observed in the choroid plexus of paclitaxel-treated rats indicates that activation of inflammatory cascades may be facilitated *directly* by the paclitaxel interaction with the choroid plexus epithelial cells. Since our *in vivo* model constrains hypothesis validity, we determined the *direct* effect of paclitaxel on Z310 cells, an *in vitro* model of the choroid plexus epithelial cells. We found that paclitaxel administration, in addition to the upregulation of TLR9 and FPR2, caused an increase in IL6 and TNF*α* levels in Z310 cells (Figure 5).

In line with our results, paclitaxel has been reported to induce pro-inflammatory cytokines in various cell lines *in vitro* (39). Regardless of the precise mechanisms involved, we can confirm an acute immune response of the choroid plexus epithelial cells to paclitaxel.

Expanding upon our observation of a significant elevation in IL6 and TNFα levels 21 days after the last injection, we hypothesized that the cause may be attributed to circulating DAMPs induced by paclitaxel treatment rather than paclitaxel itself.

Paclitaxel damages the peripheral nerve and causes the release of various cellular components (such as mtDNA and formyl peptides) into the blood, known as DAMPs (40, 41). Circulating DAMPs can reach the choroid plexus epithelial cells via the choroid plexus fenestrated capillaries and induce cytokine production (42, 43) through binding and activating their specific receptors by which proinflammatory signal transduction within the choroid plexus and then across the brain are modulated. TLR9 and FPR2 in the choroid plexus epithelial cells could respond to DAMPs and trigger innate immune responses (17, 44–49). Therefore, to simulate paclitaxel's *indirect* effects on the choroid plexus, we studied the response of TLR9 and FPR2 to their respective agonists. TLR9 responds to endogenous danger signals, such as nucleic acids, via its extensive intracellular trafficking, which could have beneficial or detrimental consequences (48, 50). mtDNA released from dying cells has been identified as a TLR9 stimulator and contains a similar structure as bacterial CpG DNA. We used CpG ODN in cell culture and determined that TLR9 agonist could promote an inflammatory reaction in Z310 cells (Figures 7A,B). This finding indicates that the choroid plexus reacts to paclitaxel products circulating in the blood, by which an inflammatory response in the brain is promoted. However, to better understand the exact mechanisms underlying TLR9 upregulation in choroid plexus epithelial cells following paclitaxel treatment, further *in vivo* pharmacological studies, such as intracisternal injection of TLR9 antagonists to modulate DAMPs signaling would be beneficial. Although we did not observe any inflammatory reactions when Z310 cells were treated with fMLP (Figures 7C,D), further investigations should be conducted to understand the lack of FPR2 response to fMLP in the Z310 cell line (51). For instance, conducting the same experiments on primary cultures of choroidal epithelial cells could provide valuable insights.

Furthermore, alteration of choroid plexus after paclitaxel treatment, including but not limited to the upregulation of TLR9 and FPR2, can activate different signaling pathways, including NF-κB, MAPK, JAK-STAT, and cAMP-mediated pathways, which can regulate the innate and adaptive immune responses (52–54), one of which could be increased leukocyte trafficking into the brain (18, 55). Entry of the immune cells into the brain and overexpression of pro-inflammatory cytokines in the CSF could activate different neuronal targets, including those involved in pain (43).

Nevertheless, while we have indicated that TLR9 and FPR2 are activated in the choroid plexus by either paclitaxel (*direct* and acute effect) or circulating DAMPs (*indirect* and chronic effect), we do not exclude the possibility that the inflammatory modulators of the periphery induced by paclitaxel may also activate TLR9 and FPR2 (*indirect* effect), as proinflammatory cytokines have been found to activate epithelial cells of the choroid plexus (21). Intense research has implicated choroid plexus immune response to peripheral inflammation induced by lipopolysaccharide to be similar to that of IL1β, IL6, and TNFα (19, 49, 52, 56, 57).

Moreover, the blood-CSF barrier is essential for maintaining CNS homeostasis and allowing immune surveillance at the CNS interfaces. As such, dysregulated choroid plexus is implicated in the progression of many neurological disorders (31). We demonstrated that involving *direct* as well as *indirect* mechanisms, paclitaxel induces immune reactivity in the choroid plexus, including an increase in pro-inflammatory cytokines levels of IL6 and TNFα (Figure 7), which could potentially alter the structure of tight junction proteins, which are essential for blood-CSF barrier functionality (31, 58). Different endogenous stimuli may be associated with blood-CSF barrier dysfunction by increasing matrix metalloproteinase secretion and downregulating genes involved in barrier maintenance (49, 56). Furthermore, TLRs activation downregulates the tight junction proteins in the choroid plexus, leading to increased barrier permeability to white blood cells (19, 30, 59, 60). Therefore, it appears imperative to underscore the alteration of the blood-CSF barrier permeability in paclitaxel treatment. In particular, the *indirect* effects of paclitaxel through DAMPs, an important and not adequately understood mechanism, can cause unprecedented chronic side effects in patients treated with paclitaxel.

Incidentally, we notably formulated the paclitaxel in Cremophor EL and 95% alcohol (1:1). The Cremophor EL is not inert and can cause toxic side effects. Correspondingly, we also observed increased TLR9 and FPR2 levels in vehicle-treated animals. This fits the fact that peripheral cytokines produced by accessory immune cells in response to stimuli, such as stress, can enter the blood in sufficiently high concentrations to reach the choroid plexus (43). As previously noted, peripheral inflammatory stimuli can definitively develop an inflammatory response in the choroid plexus (56). However, while the vehicle alone induced some degree of structural alteration, the extent and magnitude of the changes were clearly more significant in the paclitaxel-treated groups. This observation suggests that neurotoxic effects associated with paclitaxel treatment exert a more profound impact on the choroid plexus compared to the vehicle alone.

In conclusion, our timely study reveals that paclitaxel treatment can activate immunoreceptors in the choroid plexus, leading to the induction of inflammatory responses through pathways yet to be defined. We detailed how paclitaxel could, both through direct interaction with choroid plexus epithelial cells and indirectly via the release of DAMPs in peripheral blood, cause acute and chronic inflammatory responses in the choroid plexus. Following paclitaxel treatment, an inflamed choroid plexus probably could allow for the spread of inflammatory mediators into the brain via CSF and also potentially enable the infiltration of immune cells and toxins into the brain owing to compromised barrier function. Significantly, our efforts contribute towards understanding possible molecular mechanisms by which paclitaxel causes CNS neuroinflammation.

## Data Availability

The original contributions presented in the study are included in the article/Supplementary Material, further inquiries can be directed to the corresponding author.
